# Nurse-led initiation of hepatitis C care in rural Cambodia

**DOI:** 10.2471/BLT.22.288956

**Published:** 2023-02-21

**Authors:** Daniel O’Keefe, Keo Samley, Voeurng Bunreth, Tonia Marquardt, Serge Eric Bobi, Kien Antharo, Chanroeun San Kim, Hem Sothy, Thoang Sokha, Chor Samnang, Yan Sokchea, Farah Hossain, Suna Balkan, Mickael Le Paih, Jean-Philippe Dousset

**Affiliations:** a Médecins Sans Frontières–France, 205 St 132, Phnom Penh, Cambodia.; b Department of Communicable Disease Control, Ministry of Health, Phnom Penh, Cambodia.; c Battambang Provincial Health Department, Ministry of Health, Battambang, Cambodia.; d Queensland Health, Brisbane, Australia.

## Abstract

**Objective:**

To determine whether a nurse-led model of care for patients with hepatitis C virus (HCV) infections can provide safe and effective diagnosis and treatment in a resource-poor setting in rural Cambodia.

**Methods:**

The nurse-led initiation pilot project was implemented by *Médecins Sans Frontières* in collaboration with the Cambodian health ministry in two operational districts in Battambang Province between 1 June and 30 September 2020. Nursing staff at 27 rural health centres were trained to identify signs of decompensated liver cirrhosis and to provide HCV treatment. Patients without decompensated cirrhosis or another comorbidity were initiated at health centres onto combined treatment with sofosbuvir, 400 mg/day, and daclatasvir, 60 mg/day, orally for 12 weeks. Treatment adherence and effectiveness were assessed during follow-up.

**Findings:**

Of 10 960 individuals screened, 547 had HCV viraemia (i.e. viral load ≥ 1000 IU/mL). Of the 547, 329 were eligible for treatment initiation at health centres through the pilot project. All 329 (100%) completed treatment and 310 (94%; 95% confidence interval: 91–96) achieved a sustained virological response 12 weeks post-treatment. Depending on patient subgroups, this response varied from 89% to 100%. Only two adverse events were recorded; both were determined as unrelated to treatment.

**Conclusion:**

The safety and effectiveness of direct-acting antiviral medication has previously been demonstrated. Models of HCV care now need to enable greater access for patients. The nurse-led initiation pilot project provides a model for use in other resource-poor settings to scale up national programmes.

## Résumé

**Objectif:**

Déterminer si un modèle de soins dirigé par le personnel infirmier pour les patients infectés par le virus de l'hépatite C (VHC) offrirait un niveau de fiabilité et d'efficacité suffisant pour le dépistage et le traitement de la maladie dans les régions rurales du Cambodge, où les ressources sont limitées.

**Méthodes:**

Le projet pilote de prise en charge initiale par le personnel infirmier a été déployé par *Médecins Sans Frontières* en collaboration avec le Ministère cambodgien de la Santé dans deux districts opérationnels dans la province de Battambang entre le 1^er^ juin et le 30 septembre 2020. Le personnel infirmier de 27 centres de santé ruraux a été formé afin d'identifier les symptômes d'une cirrhose décompensée et fournir un traitement contre le VHC. Les patients ne présentant pas de cirrhose décompensée ni d'autre comorbidité ont entamé, dans des centres de santé, un traitement combiné au sofosbuvir (400 mg/jour) et au daclatasvir (60 mg/jour) par voie orale pendant 12 semaines. L'adhésion au traitement ainsi que son efficacité ont été évaluées durant le suivi.

**Résultats:**

Sur 10 960 individus dépistés, 547 affichaient une virémie VHC (c'est-à-dire une charge virale ≥ 1000 UI/mL). Sur ces 547 personnes, 329 étaient éligibles pour une mise sous traitement au sein d'un centre de santé dans le cadre du projet pilote. La totalité de ces 329 patients (100%) a suivi le traitement complet et 310 d'entre eux (94%; intervalle de confiance de 95%: 91–96) ont obtenu une réponse virologique durable après 12 semaines de traitement. En fonction des sous-groupes de patients, cette réponse était comprise entre 89% et 100%. Seuls deux effets indésirables ont été constatés; tous deux se sont révélés sans aucun lien avec le traitement.

**Conclusion:**

La fiabilité et l'efficacité des médicaments antiviraux à action directe ont déjà été démontrées par le passé. Les modèles de prise en charge du VHC doivent désormais garantir un meilleur accès aux patients. Le projet pilote de prise en charge initiale par le personnel infirmier offre une solution applicable à d'autres endroits où les ressources sont limitées, afin de renforcer les programmes nationaux.

## Resumen

**Objetivo:**

Determinar si un modelo de atención dirigido por el personal de enfermería para pacientes con infecciones por el virus de la hepatitis C (VHC) puede proporcionar un diagnóstico y un tratamiento seguros y eficaces en una población con pocos recursos de las zonas rurales de Camboya.

**Métodos:**

Médicos Sin Fronteras, en colaboración con el Ministerio de Sanidad de Camboya, ejecutó el proyecto piloto de tratamiento inicial dirigido por el personal de enfermería en dos distritos operativos de la provincia de Battambang entre el 1 de junio y el 30 de septiembre de 2020. El personal de enfermería de 27** **centros sanitarios rurales recibió formación para identificar los signos de cirrosis descompensada y proporcionar tratamiento contra el VHC. Los pacientes sin cirrosis descompensada u otra comorbilidad iniciaron en los centros sanitarios un tratamiento combinado con sofosbuvir, 400** **mg/día, y daclatasvir, 60** **mg/día, por vía oral durante 12** **semanas. Durante el seguimiento se evaluó la adherencia al tratamiento y su eficacia.

**Resultados:**

De las 10** **960** **personas examinadas, 547** **presentaban viremia por VHC (es decir, una concentración vírica ≥1000** **UI/ml). De los 547, 329 cumplían los requisitos para iniciar el tratamiento en los centros sanitarios a través del proyecto piloto. Los 329 (100** **%) completaron el tratamiento y 310 (94** **%; intervalo de confianza del 95** **%:** **91-96) lograron una respuesta virológica sostenida 12** **semanas después del tratamiento. Dependiendo de los subgrupos de pacientes, esta respuesta varió entre el 89** **% y el 100** **%. Solo se registraron dos acontecimientos adversos; en ambos casos se determinó que no estaban relacionados con el tratamiento.

**Conclusión:**

La seguridad y eficacia de los antivíricos de acción directa ya ha quedado demostrada. Ahora es necesario que los modelos de atención al VHC permitan un mayor acceso a los pacientes. El proyecto piloto de tratamiento inicial dirigido por el personal de enfermería ofrece un modelo que se puede utilizar en otras poblaciones con pocos recursos para ampliar los programas nacionales.

## ملخص

**الغرض:**

تحديد ما إذا كان بإمكان نموذج الرعاية الذي تقوده الممرضات للمرضى المصابين بفيروس التهاب الكبد الوبائي ج، توفير التشخيص والعلاج الآمن والفعال في بيئة فقيرة الموارد في ريف كمبوديا.

**الطريقة:**

نفذت منظمة أطباء بلا حدود المشروع التجريبي للخطوات الأولية بقيادة الممرضات بالتعاون مع وزارة الصحة الكمبودية في منطقتين تشغيليتين في مقاطعة باتامبانج في الفترة بين 1 يونيو/حزيران و30 سبتمبر/أيلول لعام 2020. تم تدريب أطقم التمريض الموجودة في 27 مركزًا صحيًا ريفيًا على تحديد علامات تليف الكبد اللا تعويضي وتوفير علاج لفيروس التهاب الكبد الوبائي ج (HCV). بدأ المرضى غير المصابين بتليف الكبد اللا تعويضي، أو اعتلال مشترك آخر، في المراكز الصحية العلاج المشترك المكون من سوفوسبوفير بجرعة 400 مللي جرام/يوميًا، وداكلاتازفير بجرعة 60 مللي جرام/يوميًا عن طريق الفم لمدة 12 أسبوعًا. وتم تقييم الالتزام بالعلاج وفعاليته أثناء المتابعة.

**النتائج:**

من بين 10960 فردًا تم فحصهم، كان هناك 547 فردًا مصابًا بفيروس التهاب الكبد الوبائي ج (HCV) في الدم (أي الحمل الفيروسي ≥ 1000 وحدة دولية/مل). من بين 574 فردًا، كان هناك 329 شخصًا مؤهلًا لبدء العلاج في المراكز الصحية من خلال المشروع التجريبي. أكمل 329 شخصًا (%100) العلاج وحقق 310 شخصًا (%94؛ %95 بفاصل ثقة: من 91 إلى 96) استجابة فيروسية مستدامة بعد مرور 12 أسبوعًا من العلاج. اعتمادًا على مجموعات المرضى الفرعية، تراوحت هذه الاستجابة بين %89 و%100. لم يتم تسجيل سوى حالتين سلبيتين، وتم تحديد كلاهما على أنهما لا علاقة لهما بالعلاج.

**الاستنتاج:**

اتضح مسبقًا سلامة الأدوية المضادة للفيروسات ذات المفعول المباشر وفعاليتها. تحتاج نماذج رعاية فيروس الكبد الوبائي ج (HCV) الآن إلى إمكانية وصول أكبر للمرضى. ويوفر مشروع الخطوات الأولية التجريبي بقيادة الممرضات نموذجًا لاستخدامه في البيئات الأخرى التي تفتقر إلى الموارد لتوسيع نطاق البرامج الوطنية.

## 摘要

**目的:**

旨在确定针对丙型肝炎病毒 (HCV) 感染患者的以护士为主导的护理模式是否能够在柬埔寨农村资源匮乏地区提供安全有效的诊断和治疗。

**方法:**

*无国界医生组织（独立人道医疗救援组织）*与柬埔寨卫生部于 2020 年 6 月 1 日至 9 月 30 日期间在马德望省的两个试点地区合作开展了以护士为主导的试点项目。27 个农村卫生中心的护理人员接受了关于识别失代偿期肝硬化迹象和提供 HCV 治疗方面的培训。未出现失代偿期肝硬化或其他合并症的患者在卫生中心接受口服索非布韦（400 毫克/天）和达卡他韦（60 毫克/天）的联合治疗，治疗持续 12 周。在随访期间评估了治疗依从性和疗效。

**结果:**

在 10,960 个被筛查个体中，有 547 个人患有 HCV 病毒血症（即，病毒载体 ≥ 1000 IU/mL）。在这 547 人中，有 329 人有资格通过试点项目在保健中心进行治疗。这 329 (100%) 人均完成了治疗，其中 310 人（94%；95% 置信区间：91–96）在治疗后 12 周内获得持续病毒学应答。根据患者亚组的不同，应答率在 89% 到 100% 之间不等。仅记录有 2 例不良事件；均被确定与治疗无关。

**结论:**

直接作用的抗病毒药物的安全性和有效性已得到证实。现在需要进一步普及 HCV 护理模式，以便惠及更多患者。以护士为主导的试点项目提供了一种可用于其他资源匮乏地区的护理模式，有利于进一步扩大国家项目。

## Резюме

**Цель:**

Определить, может ли модель ухода за пациентами с инфекцией вируса гепатита С (ВГС) под руководством медсестер обеспечить безопасную и эффективную диагностику и лечение в условиях ограниченных ресурсов в сельской местности Камбоджи.

**Методы:**

В период с 1 июня по 30 сентября 2020 года организация *Врачи без границ* (*Médecins Sans Frontières*) в сотрудничестве с Министерством здравоохранения Камбоджи реализовала пилотный проект по началу лечения под руководством медсестер в двух работоспособных районах провинции Баттамбанг. Сестринский персонал 27 сельских медицинских учреждений прошел обучение по выявлению признаков декомпенсированного цирроза печени и лечению ВГС. Пациенты без декомпенсированного цирроза печени или других сопутствующих заболеваний начинали в медицинских учреждениях комбинированную терапию препаратами «Софосбувир» в дозе 400 мг/день и «Даклатасвир» в дозе 60 мг/день перорально в течение 12 недель. Соблюдение режима лечения и его эффективность оценивали во время последующего наблюдения.

**Результаты:**

Из 10 960 человек, прошедших скрининг, у 547 была выявлена ВГС-вирусемия (то есть вирусная нагрузка ≥ 1000 МЕ/мл). Из 547 человек 329 были допущены к началу лечения в медицинских учреждениях в рамках пилотного проекта. Все 329 человек (100%) завершили лечение, и 310 человек (94%; 95%-й ДИ: 91–96) достигли устойчивого вирусологического ответа через 12 недель после лечения. В зависимости от подгрупп пациентов этот ответ варьировался от 89 до 100%. Было зарегистрировано только два нежелательных явления. Оба были определены как не связанные с лечением.

**Вывод:**

Безопасность и эффективность противовирусных препаратов прямого действия была продемонстрирована ранее. Модели лечения ВГС теперь должны предоставлять более широкий доступ для пациентов. Пилотный проект по началу лечения под руководством медсестер представляет собой модель для использования в других условиях с ограниченными ресурсами для расширения масштабов государственных программ.

## Introduction

Given the demonstrated safety and effectiveness of current, direct-acting antiviral treatments for hepatitis C virus (HCV) infection and the validity of rapid, point-of-care tests,^1^^,^^2^ international discussions and investigations are now focusing on the best models of care for HCV-infected patients. Several implementation studies have evaluated simplified models of HCV care provided in decentralized settings that involve substantial task-shifting to less-specialized staff (e.g. treatment initiation and follow-up by nursing staff).^3^^–^^6^ Furthermore, innovations in rapid, point-of-care testing have enabled same-day diagnosis and, potentially, same-day treatment initiation.^1^^,^^4^^,^^7^ Importantly, the ability to decentralize HCV testing and treatment facilitates the implementation of a so-called one-stop-shop model of HCV care, whereby diagnosis, treatment and follow-up are provided at a single location, with specialist referral for patients with cirrhosis.^4^^,^^8^

Nurse-led models of HCV treatment have been implemented internationally in a variety of contexts, including community, hospital and custodial settings.^9^^–^^12^ However, these have been in high-income countries with developed HCV elimination programmes, and all included countries on track to achieve the World Health Organization’s (WHO’s) global targets for eliminating HCV.^13^ Evidence is still required on the effectiveness of nurse-led models of direct-acting antiviral treatment initiation in low- and middle-income countries.

For Cambodia, there are no robust estimates of the prevalence of HCV infection, which historically was mainly iatrogenic. However, recent geographical and subpopulation seroprevalence estimates range from 2.6% to 14.7%.^5^^,^^14^ Access to HCV testing and treatment is limited across the country.^15^ Since 2016, *Médecins Sans Frontières* has implemented HCV testing and treatment projects in: (i) urban Phnom Penh, using a progressively simplified care model in a government hospital; and (ii) rural areas, involving basic primary health outposts with non-specialist staff.^5^^,^^6^ In 2020, *Médecins Sans Frontières* expanded on these projects: the nurse-led initiation pilot project was implemented to evaluate pre-treatment assessment, treatment initiation and follow-up by nursing staff in a resource constrained rural setting in Battambang Province, Cambodia. The pilot project involved minimal resources – there were no medical doctors on-site in health centres and no transient elastography machines for liver assessment, and laboratories did not have the capacity to perform some pre-treatment assessments, such as deriving aspartate aminotransferase-to-platelet ratio index scores.

The aim of this paper was to report the outcomes of the nurse-led initiation pilot project, including patient retention, treatment effectiveness and adverse events. In addition, the HCV testing and treatment model of care is described; and the effectiveness of the project is evaluated in terms of patient linkage to care, patient retention in treatment, the occurrence of adverse patient outcomes and HCV cure rates.

## Methods

The nurse-led initiation pilot project was implemented between 1 June and 30 September 2020 in two operational districts in Battambang Province: Sangke and Thmar Kaul. In 2019, Sangke operational district had a population of 173 000 across 16 communes and 105 villages; Thmar Kaul operational district had a population of 183 000 across 17 communes and 161 villages.^16^ Each district has two referral hospitals. In addition, Sangke operational district has 15 rural health centres and Thmar Kaul operational district has 18. These rural health centres are basic primary health posts run by non-specialist nursing staff who provide services such as basic health screening, antenatal care, tuberculosis screening and treatment, childhood immunization and human immunodeficiency virus (HIV) testing. Ten rural health centres in Sangke operational district and 17 in Thmar Kaul participated in the pilot project, with at least two government nurses supporting the project at each health centre but no on-site medical doctors. Before project implementation, health centre staff attended two days’ training on HCV screening and treatment and on identifying signs of decompensated liver cirrhosis and other comorbidities. Staff received ongoing supervision from *Médecins Sans Frontières* at least once a month to ensure practice quality and the accuracy of data collection.

Ethical approval was provided by the Cambodian National Ethics Committee for Health Research (Project No. 076) and the project was compliant with the provisions of the Declaration of Helsinki.^17^

### HCV screening

Individuals were screened voluntarily for HCV infection, regardless of previous HCV treatment, if they were: (i) 18 years or older; (ii) not a person living with HIV; (iii) not pregnant or breastfeeding; (iv) not displaying symptoms of tuberculosis or receiving tuberculosis treatment (though they could take part in screening once treatment ended); and (v) not currently receiving HCV treatment. Cases were found both actively and passively (i.e. individuals presented to rural health centres). Active case-finding involved a team of existing village health volunteers who visited locations such as villages, pagodas (i.e. temples) and schools, along with *Médecins Sans Frontières* staff, to perform rapid HCV-antibody testing and to refer patients who tested positive to the pilot project for confirmatory testing.

**Fig. 1:**
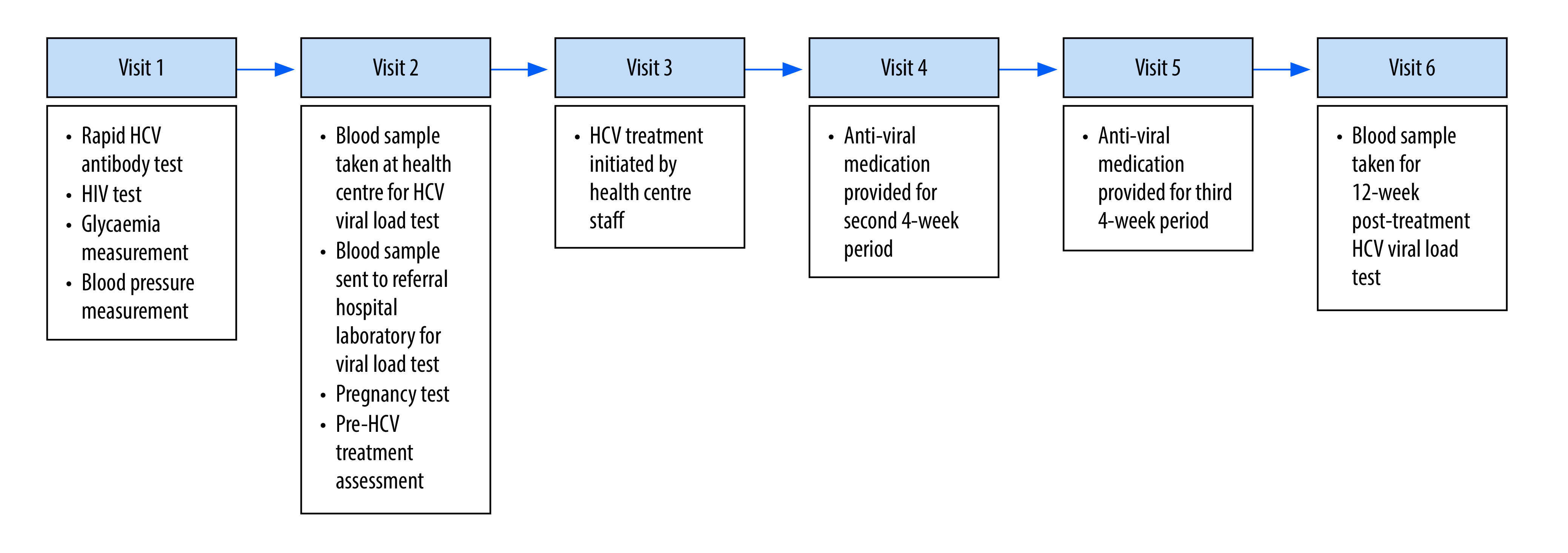
Health centre visits, nurse-led hepatitis C care pilot project, Cambodia, 2020

HCV: hepatitis C virus; HIV: human immunodeficiency virus.

Notes: Only patients without signs of decompensated liver cirrhosis or another comorbidity initiated treatment at a health centre; others were referred to hospital. Patients initiated onto treatment at a health centre were prescribed combined sofosbuvir, 400 mg/day, and daclatasvir, 60 mg/day, orally daily for 12 weeks.

Following pretest counselling, HCV-antibody serology was performed using a rapid diagnostic test (SD Bioline HCV, Abbott, Abbott Park, United States of America; visit 1 in Fig. 1). If the result was positive, patients were offered HIV, glycaemia and blood pressure assessments. Patients who tested positive for HIV were referred to a government-run HIV clinic, where they could receive HCV treatment. Patients with a glycaemia measurement of 200 mg/dL or higher and those with a blood pressure measurement of 140/90 mmHg or higher were referred to a chronic diseases clinic at the referral hospital or to a private hospital, but remained eligible for HCV care via the pilot project. Because of cold chain considerations, HCV viral load blood samples were taken only once a week at health centres before transport to testing laboratories. Patients who tested positive for HCV antibodies were given an appointment for the next available occasion for venous blood sampling. All patients seen at health centres were asked to pay 1 United States dollar (US$) for HCV screening, except those with a government poor card.

### Pre-treatment assessment

To expedite treatment initiation, pre-treatment assessment was performed prospectively (Fig. 1). Signs of potential decompensated liver cirrhosis were investigated during the appointment for the venous blood sample needed to assess HCV viral load, before blood was drawn. Patients were assessed clinically and asked about their past or current experience of the following symptoms of decompensated cirrhosis: (i) jaundice; (ii) encephalopathy; (iii) gastrointestinal bleeding; (iv) ascites; and (v) oedema.^18^ In addition, each patient’s general condition was assessed by standard clinical examination (including blood pressure, pulse and temperature measurements) and details were recorded of any previously diagnosed chronic disease with ongoing treatment and any previous HCV treatment (either direct-acting antiviral or pre-direct-acting antiviral treatment).

If any symptom of decompensated cirrhosis was present, the patient was referred to the referral hospital. Consequently, only patients without decompensation or another specified complication were treated in the pilot project. For patients with compensated cirrhosis in a country where the prevalence of HCV genotype 3 is under 5%, such as Cambodia,^19^^,^^20^ WHO guidelines recommend 12-week treatment with sofosbuvir and daclatasvir, which means the pilot project’s treatment regimen was sufficient to treat compensated cirrhosis.

Pregnancy testing was performed in women aged between 18 and 50 years and a venous blood sample was taken only from women returning a negative pregnancy test result. Blood samples were transported on the day of sampling to the Thmar Kaul or Sangke referral hospital laboratory, where HCV viral load was assessed using the GeneXpert^®^ HCV Viral Load test (Cepheid, Sunnyvale, USA), with HCV viraemia defined as an HCV viral load of 1000 IU/mL or higher.^19^ Patients received results on the same day and health centre staff arranged an appointment for treatment initiation, if required. In addition, blood from patients with HCV viraemia was also tested for hepatitis B virus (HBV) surface antigen at the referral hospital. Subsequently, patients with an HBV coinfection were referred to the hospital for treatment.

### HCV treatment

At the start of treatment, health centre staff provided patients with information about treatment adherence and potential side-effects. Those taking a proton pump inhibitor (e.g. omeprazole) were advised to stop during direct-acting antiviral treatment, unless it was on medical prescription. Women younger than 50 years were offered family planning services before antiviral treatment.

All patients were treated with combined sofosbuvir, 400 mg/day, and daclatasvir, 60 mg/day, orally for 12 weeks. At treatment initiation, patients received 28 days of medication, with instructions to return to the health centre after 1 and 2 months for further doses (total treatment duration: 84 days). Treatment was provided at no cost.

When patients returned to the health centre, treatment adherence was assessed from patients’ reports and returned medication boxes. In addition, each patient’s clinical condition was re-assessed. If any side-effects were experienced during treatment, the patient was referred to the referral hospital. Following consultation, further medication doses were provided. At the consultation after the second month of treatment, an appointment was made for a blood sample to be taken to measure the HCV viral load 12 weeks after the end of treatment, to assess the treatment outcome. A cure was defined as an HCV viral load 12 weeks post-treatment less than 1000 IU/mL (i.e. a sustained virological response) and a higher load was regarded as treatment failure. Patients who experienced treatment failure were referred to a referral hospital for medical consultation to identify the cause.

### Project evaluation

All patient data were initially recorded using a paper template, then manually entered into an electronic medical record system using REDCap software (Vanderbilt University, Nashville, USA). Data were included on all patients who were screened or initiated treatment at any of the 27 participating rural health centres in Sangke or Thmar Kaul operational districts during the pilot project. Assessment of HCV viral load 12 weeks post-treatment was an essential part of the treatment protocol. However, after the pilot project, such testing was optional, in line with Cambodian national guidelines.^21^

#### Data analysis

A descriptive analysis compared the percentages of patients with specific HCV viral load test results in different sex, age and presenting operational district (i.e. Thmar Kaul or Sangke) categories using the χ2 test for categorical variables. Patients were considered to have completed treatment if they collected their third 4-week supply of antiviral medication, thereby finishing the 12-week course. The percentage of patients who achieved a sustained virological response was calculated in: (i) an intention-to-treat analysis that included all patients who initiated treatment at a health centre during the pilot period, with every outcome other than a cure considered a failure; and (ii) a modified intention-to-treat analysis that included only patients whose HCV viral load was assessed 12 weeks post-treatment.^4^ The Clopper-Pearson method was used to calculate 95% confidence intervals (CIs).^6^ Other treatment outcomes included adverse events such as death, loss to follow-up and the medical decision to stop HCV treatment. The data analysis was performed using Stata/SE version 17.0 (StataCorp LLC, College Station, USA).

## Findings

### Screening

**Fig. 2:**
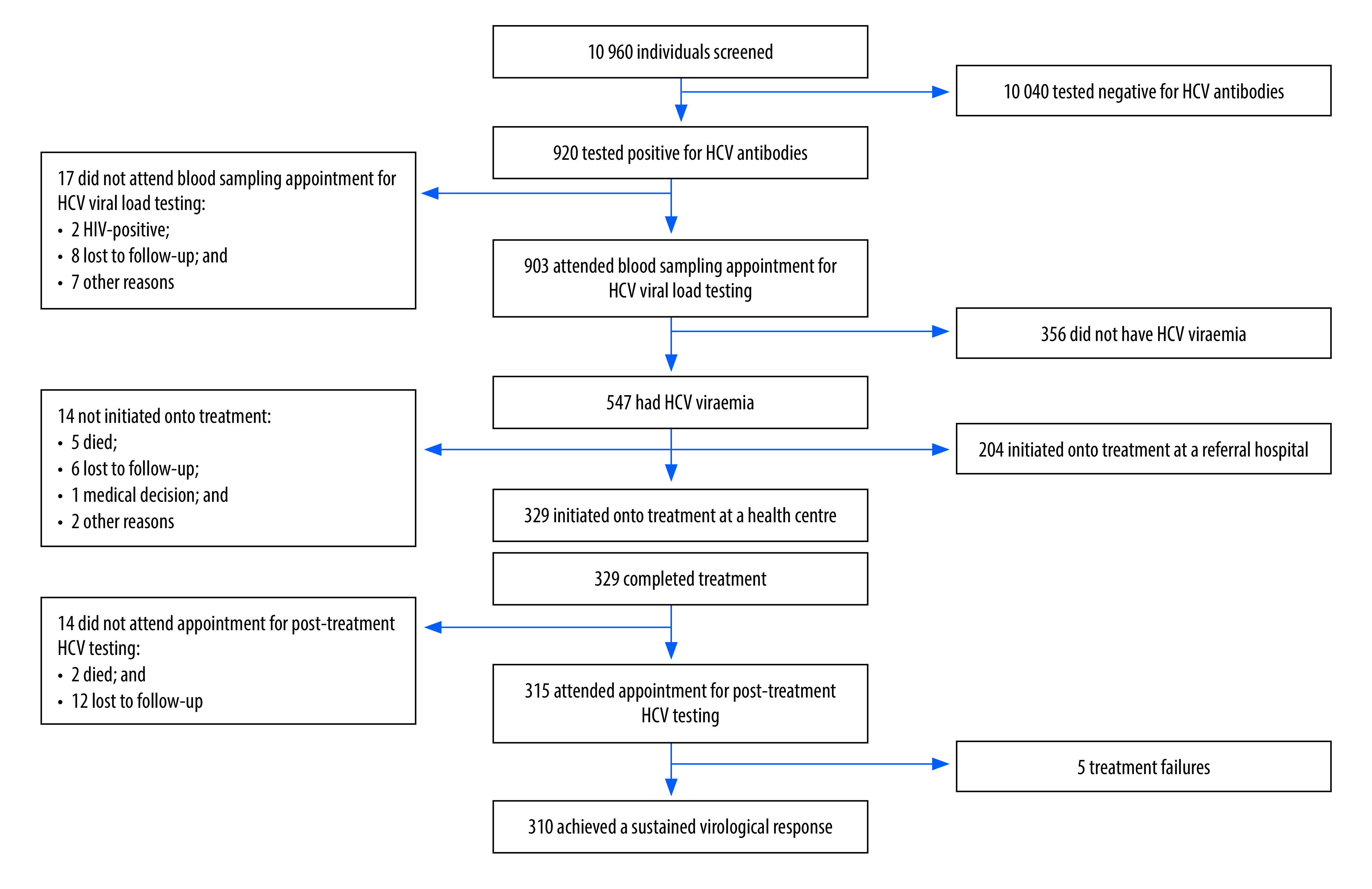
Screening, testing, treatment and outcomes, nurse-led hepatitis C care pilot project, Cambodia, 2020

HCV: hepatitis C virus; HIV: human immunodeficiency virus.

Notes: Only patients without signs of decompensated liver cirrhosis or another comorbidity initiated treatment at a health centre; others were referred to hospital. Patients initiated onto treatment at a health centre were prescribed combined sofosbuvir, 400 mg/day, and daclatasvir, 60 mg/day, orally daily for 12 weeks. Hepatitis C virus (HCV) viraemia was defined as an HCV viral load ≥ 1000 IU/mL and a sustained virological response was defined as an HCV viral load < 1000 IU/mL 12 weeks post-treatment.

During the pilot project period, 10 960 individuals were screened for HCV infection: 4704 (43%) in Sangke operational district and 6256 (57%) in Thmar Kaul operational district. No registry data were recorded for individuals with a negative HCV-antibody test result. In total, 920 patients had a positive HCV-antibody test result (Table 1; Fig. 2). Seventeen (2%) of the 920 who tested positive did not attend their blood sample appointment. Of the 903 patients who did attend (98% of those with a positive HCV-antibody test result), 61% (547/903) had HCV viraemia. There was a significant difference between males and females in the percentage diagnosed with HCV viraemia (*P* = 0.01). The percentage diagnosed with HCV viraemia was not significantly different when comparing age groups or presenting operational districts (Table 1). The median turn-around time between HCV-antibody screening and blood sampling for viral load assessment was 0 days (interquartile range, IQR: 0–5), which means that most patients underwent HCV-antibody testing and gave a blood sample for viral load testing on the same day.

**Table 1:** Hepatitis C virus test results, nurse-led hepatitis C care pilot project, Cambodia, 2020

Sociodemographic variable	No. patients with a positive HCV-antibody test result (%)			*P* ^a^
	All (*n* = 920)	Without viraemia^b,c^ (*n* = 356)	With viraemia^b,c^ (*n* = 547)	
**Sex**				0.01
Male	367 (40)	124 (35)	235 (43)	
Female	553 (60)	232 (65)	312 (57)	
**Age, in years **				0.87
≤ 44	183 (20)	69 (19)	113 (21)	
45–54	168 (18)	63 (18)	104 (19)	
55–64	369 (40)	148 (42)	214 (39)	
≥ 65	200 (22)	76 (21)	116 (21)	
**Operational district^d^**				0.43
Sangke	365 (40)	148 (42)	213 (39)	
Thmar Kaul	555 (60)	208 (58)	334 (61)	

HCV: hepatitis C virus.

^a^ *P*-values are for the difference between sociodemographic subgroups and hepatitis C virus (HCV) viral load status.

^b^ HCV viraemia was defined as an HCV viral load ≥ 1000 IU/mL.

^c^ Seventeen patients did not attend their blood sample appointment for viral load testing.

^d^ The study was performed in two operational districts in Battambang Province, Cambodia.

### Treatment and treatment retention

Of the 547 patients with HCV viraemia, 14 (2.6%) did not start direct-acting antiviral treatment (Fig. 2). Of the remaining 533 patients (97% of those with viraemia) who all initiated treatment, 38% (204/533) were referred to a referral hospital for treatment initiation, primarily due to a possible comorbidity, including liver decompensation. The other 62% (329/533) were initiated onto treatment at a health centre (Fig. 2). The median turn-around time between the HCV viral load testing date and the appointment for initiating treatment was 8 days (IQR: 6–12). One patient who started treatment at a health centre during the pilot project was ultimately followed up at a referral hospital after missing some treatment doses. Nevertheless, this patient was included in the pilot project cohort.

### Treatment outcomes

All 329 patients (100%) who initiated treatment at a health centre in the pilot project completed the treatment course. Of patients who initiated treatment at a referral hospital, 96% (196/204) completed treatment. Fourteen of the 329 (4%) did not return for 12-week post-treatment testing: (i) two died (one by stroke and one by unknown causes; both determined to be unrelated to their direct-acting antiviral treatment); and (ii) 12 were lost to follow-up. Consequently, 315 patients (96%) completed treatment and returned for 12-week post-treatment testing, among whom 310 (98%) achieved a sustained virological response and five (2%) experienced treatment failure (Fig. 1).

In the intention-to-treat analysis, 310 of the 329 patients initiated onto treatment (94%; 95% CI: 91–96) achieved a sustained virological response and 19 (6%) did not (14 did not return for post-treatment testing and five experienced treatment failure). In the modified intention-to-treat analysis, which included only the 315 patients who returned for 12-week post-treatment testing, 310 (98%; 95% CI: 96–99) achieved a sustained virological response and five (2%) did not.

The percentage of patients with a sustained virological response in different subgroups is shown in Table 2. In the intention-to-treat analysis, the percentage across subgroups ranged from 89% to 100%. The lowest percentage was in patients aged 44 years or younger, at 89% (95% CI: 81–94) – a disproportionately large number in this subgroup was reported lost to follow-up before post-treatment testing. In the modified intention-to-treat analysis, the percentage of patients with a sustained virological response ranged from 97% to 100% across subgroups.

**Table 2:** Sustained virological responses, nurse-led hepatitis C care pilot project, Cambodia, 2020

Sociodemographic variable	No. patients initiating HCV treatment at a health centre^a^ (%)	No. patients whose viral load was assessed 12 weeks post-treatment (%)	No. patients with a sustained virological response^b^ (%; 95% CI)	
			Intention-to-treat analysis (*n* = 329)	Modified intention-to-treat analysis^c^ (*n* = 315)
**All patients**	329 (100)	315 (100)	310 (94; 91–96)	310 (98; 96–99)
**Sex**				
Male	142 (43)	137 (43)	136 (96; 91–98)	136 (99; 96–100)
Female	187 (57)	178 (57)	174 (93; 88–96)	174 (98; 94–99)
**Age, in years**				
≤ 44	99 (30)	91 (29)	88 (89; 81–94)	88 (97; 91–99)
45–54	70 (21)	68 (22)	67 (96; 88–99)	67 (99; 92–100)
55–64	114 (35)	110 (35)	109 (96; 90–99)	109 (99; 95–100)
≥ 65	46 (14)	46 (14)	46 (100; 92–100)	46 (100; 92–100)
**Operational district^d^**				
Sangke	104 (32)	100 (32)	98 (94; 88–98)	98 (98; 93–100)
Thmar Kaul	225 (68)	215 (68)	212 (94; 90–97)	212 (99; 96–100)

CI: confidence interval; HCV: hepatitis C virus.

^a^ Only patients without signs of decompensated liver cirrhosis or another comorbidity initiated treatment at a health centre; others were referred to hospital.

^b^ A sustained virological response was defined as a hepatitis C virus (HCV) viral load < 1000 IU/mL 12 weeks after the end of treatment.

^c^ The modified intention-to-treat analysis included only patients who completed post-treatment HCV viral load testing 12 weeks after the end of treatment.

^d^ The study was performed in two operational districts in Battambang Province, Cambodia.

## Discussion

The nurse-led initiation pilot project evaluated a novel, real-world model of HCV testing and treatment that involved patients being assessed and initiated onto direct-acting antiviral treatment by nursing staff in a resource-poor setting. The pilot project demonstrated high levels of patient retention and treatment effectiveness, with few adverse events.

Implementation presented several challenges, the principal of which was limited screening coverage, especially among individuals older than 45 years, who were known to be particularly at risk of historic HCV infection in Cambodia. To increase awareness of screening, information, education and communication activities targeting prospective participants were conducted at health centres, and project staff collaborated with local organizations. In addition, health volunteers actively looked for cases in catchment villages. There were also challenges with the procurement and management of project stock, such as test cartridges and medications. Importantly, however, the pilot project was strongly supported by the Cambodian government and project activities were easily absorbed into day-to-day health centre work. We estimated the cost of treatment per patient was US$ 360 by taking into account the costs of: (i) managing *Médecins Sans Frontières* support office and project staff (including local and foreign staff); (ii) procurement of tests and treatments; (iii) logistics; (iv) case-finding activities; and (v) other costs. This amount is higher than it would be for a future government programme, which would not involve many of the overhead costs associated with the pilot project.

For the pilot project, *Médecins Sans Frontières* provided funding for diagnostic equipment, antiviral treatment and the transportation of blood samples to hospital laboratories. The utilization of existing government infrastructure meant that additional costs were quite small, which indicates that the care model is potentially sustainable both in Cambodia and in other countries replicating our approach. Since project completion, *Médecins Sans Frontières* has collaborated with the Cambodian government to train staff and implement the nurse-led initiation model of care across 10 further operational districts in six provinces. Additionally, preliminary results from this project were included in newly updated WHO guidelines as a case study of how HCV care models may be further simplified, which is testament to the model’s feasibility.^22^

The project had several limitations. First, sustained virological responses in patients initiating treatment at a referral hospital were not always assessed because national HCV guidelines stipulate that testing is optional.^21^ Consequently, patients treated at rural health centres could not be compared with those treated at referral hospitals. Second, although this *Médecins Sans Frontières*-supported project benefited from a level of infrastructure that is not always available in resource-poor settings, it was implemented in existing government health centres by government staff, which means that any additional resources required could be provided in comparable settings. Third, patients may have felt compelled to attend HCV services because testing and treatment were provided at no cost in the pilot project (aside from the US$ 1 screening fee); otherwise they may have been disinclined to attend because of co-payments required by other Cambodian health-care providers.^23^

With the advent of direct-acting antivirals, the global elimination of HCV infection has become a reality.^1^ However, few countries are on track to meet elimination targets.^24^ In many places, HCV treatment may be inaccessible because it is provided only in tertiary or specialist settings after multiple consultations and on referral, and because out-of-pocket costs are high.^25^^,^^26^ Although nurse-led models of HCV care have previously been implemented and evaluated internationally,^9^^–^^11^ our nurse-led initiation pilot project is innovative because of its context: project nurses were working in extremely resource-poor settings in a low-income country. After two days’ training and with ongoing supervision, health centre staff were able to: (i) assess the presence of liver decompensation and other comorbidities; (ii) start HCV treatment quickly in patients without complications; and (iii) achieve very high rates of treatment completion and cure. Moreover, infrastructure needs were minimal, without transient elastography machines or pre-treatment assessment (e.g. aminotransferase-to-platelet ratio index scores). The project’s outcomes reflect the competency of nursing staff (and health centre staff more broadly), who were working within many constraints, and also the safety and effectiveness of current direct-acting antivirals. Our findings confirm that HCV care provided by less-specialized staff in a community-based setting can produce high retention and cure rates.^4^^,^^6^^,^^27^

Our findings illustrate several aspects of existing nurse-led practice. First, there is increasing evidence that performing HCV testing and treatment using a decentralized and integrated approach involving task-shifting can be a safe and effective way of achieving a diagnosis and cure.^28^ Second, ample research demonstrates that care quality, patient safety and the acceptability of care are not diminished by nurses having an expanded role.^29^ Such expanded roles are manifested in the nurse practitioner, who is qualified to screen, diagnose and prescribe medications for various acute and chronic conditions.^30^ In many settings around the world, nurse practitioners treat and follow up people living with an HCV infection, thereby expanding access to HCV care.^11^^,^^31^ Internationally, nursing staff are increasingly adopting roles traditionally performed by physicians.^29^ In many countries where a shortage of health-care providers remains a barrier to accessing HCV care, integrating care within primary care services, with task-shifting to less-specialized staff, can help fill the gap.^32^ This approach emphasizes person-centred care, as illustrated by the nurse-led initiation pilot project, in which HCV testing and treatment were provided close to where people live and involved services that were highly acceptable to, and deliberately minimized the burden on, patients. Even so, it should be remembered that nursing staff in the project were supported by general practitioners at partnered referral hospitals who assessed patients with decompensated cirrhosis or another comorbidity. In fact, we would like to emphasize the importance of the role played by specialist and non-specialist clinicians in supporting nurse-led HCV testing and treatment.^8^

Given that few WHO Member States are on track to achieve global HCV elimination targets,^13^ there is a clear need to scale up testing, reduce the number of diagnostic and assessment tests and decentralize treatment.^1^^,^^2^^,^^6^ This need is reflected in international recommendations.^33^ Research into the effectiveness of novel models of HCV care delivery can increase the evidence base on what is possible and effective generally and can guide other, similar interventions in low-income, low-resourced settings, which is where interventions and innovation are needed most. Our nurse-led initiation pilot project is an exemplar of a model of care that demonstrates how direct-acting antiviral treatment can be provided safely and effectively in a resource-poor setting. It is an approach that could be emulated, especially in similar rural, primary health-care facilities worldwide.
